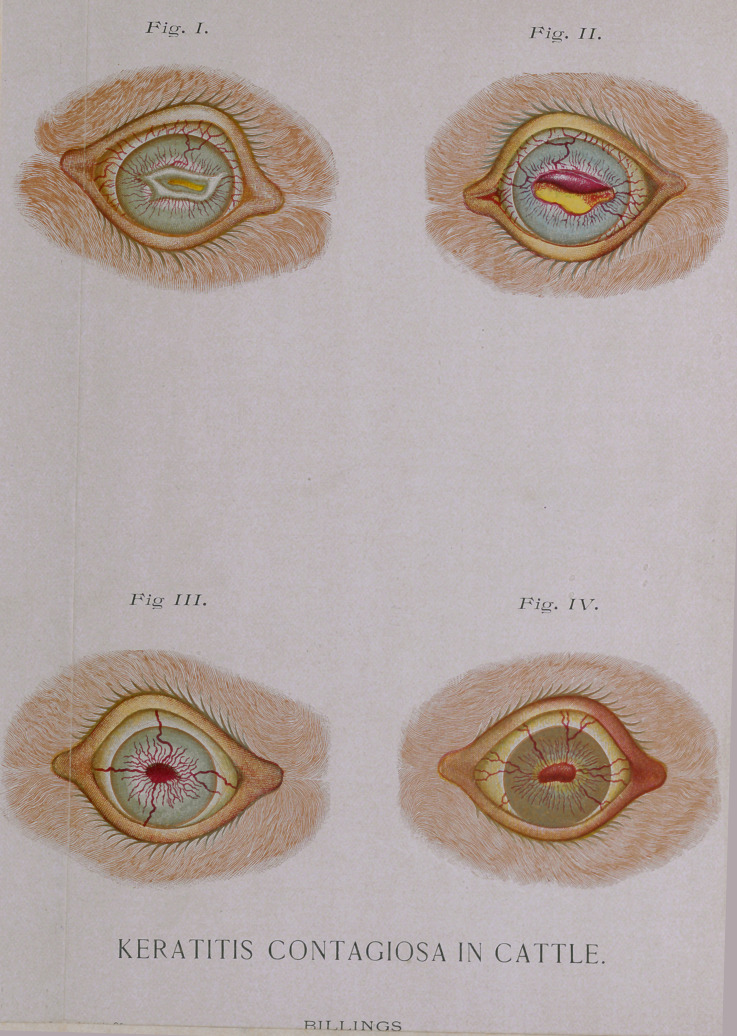# Keratitis Contagiosa in Cattle

**Published:** 1889-04

**Authors:** Frank S. Billings

**Affiliations:** Director of the Patho-Biological Laboratory of the State University of Nebraska; Lincoln, Nebraska


KERATITIS CONTAGIOSA IN CATTLE.

BILLINGS

THE

Buffalo Medical and Surgical Journal

VOL. XXVIII. APRIL, 1889. No. 9

### Original Communications.

### *KERATITIS CONTAGIOSA IN CATTLE.*

BY FRANK S. BILLINGS,
Director of the Patho-Biological Laboratory of the State University of Nebraska.

This is not a new disease by any means, so far as the United States are concerned, nevertheless I have been unable to find any description of it in the literature at my command. While new to myself until the past Summer, there have been quite a number of reports of its existence, and complaints about it, from farmers and breeders of cattle in some of the live-stock journals of our western States. Under these circumstances, it would seem that a description of its clinical phenomena and gross pathological lesions may not be without scientific interest to the opthalmologist, and have some practical value as well, especially as experience has shown that the extension of the disease over the members of a herd of cattle can be easily prevented by isolation measures, and its course much shortened by the mildest and simplest therapeutic treatment.

*History.*—During the past year, three quite extensive outbreaks have been reported to me in Nebraska; one having been at Kearney, and another at Gibbon, in Buffalo county, while a third occurred in the immediate vicinity of Lincoln, thus giving an opportunity for some personal observations. Of the outbreak at Kearney, the owner wrote me that "the trouble appears to begin as a small spot on the eyeball, the eye running and gradually growing worse, showing a purplish color, and becoming very sore; the pupil seems to protrude as though proud-flesh, or something of that nature, grew in the center." The above will be found to be an unusually good description from the hands of a layman.

The disease first appeared, in the vicinity of Lincoln, in a herd of dairy cows, about July 1, 1888. Its extension over them was very slow indeed, and although there were several horses among the cattle, or exposed to more or less danger of infection, the disease did not extend to them, nor to the men milking and caring for the animals. Up to October 1st, ten cows, out of about seventy-five, and seventeen calves had become affected, at which time my observations had to cease on account of important engagements elsewhere.

*Clinical Phenomena and Gross Pathological Lesions.*—The disease first manifests itself by the discharge of a thin, clear, watery fluid from the conjunctival sac; marked photophobia is an early symptom; the eyelids being closed and somewhat swollen, though the afflicted animal can open them easily enough if startled, and has complete control over their movements; the discharge rapidly increases in quantity, the conjunctiva becoming more and more swollen, until, in severe cases, the engorgement of the vessels becomes so intense that its general color is almost a diffuse copper-red (this has not been shown in the accompanying illustrations, on account of the unfortunate necessity of all possible economy in the number of cuts[1]). In many severe cases, the discharge becomes purulent. While a careful examination of the diseased animals has shown that the rise in temperature is but very slight, still they present phenomena which the casual observer might mistake for those of high fever. Their heads are held depressed, the ears become pendulant, they refuse to eat, and rapidly emaciate, while the yield of milk lessens materially. These conditions augment during the first eight or ten days, the photophobia correspondingly increasing; instead of fever, they must be attributed to the severe pain which the animal is suffering; intra-ocular pressure is present in an excessive degree, but the cause thereof is entirely to be sought in a marked increase in the quantity of fluid in the anterior chamber of the eye, the cornea of which becomes distended and very

1. These are state reasons, and have no reference to this journal.—Ed.

prominent. While the disease has never been reported to me as beginning in both eyes at one and the same time, still, in almost all cases, it has been noticed to extend to the other eye.

At about the second or third day from the time the first eye has been observed to be affected, a very delicate cloudiness makes its appearance at or near the center of the eye, which continually increases, the membrane becoming thicker and thicker, and more and more opaque, which conditions gradually extend to the sclerotic edges. This center is at first of a pearly white color, and in some cases may not become more than a creamy white, being much thickened, but in many a small yellowish speck will be seen to form, which gradually becomes larger, the tissues over it becoming thinner and thinner. This yellowish spot will be seen to become surrounded by a wall of thick, swollen, indurated tissue, of a white color, while outside of this will be a more or less pearly white substance, losing itself in a bluish-white tissue as the peripheries of the cornea are approached. See Fig. 1. (It will be self-evident that all these fine points of shading could not be illustrated to perfection except at great expense; hence the reader must make due allowance for illustrations in which it has only been endeavored to show the essential points.) From this indurated tissue, which encloses the apostematous center, may be seen numerous delicate blood-vessels, taking their course in a serpentine manner towards the sclerotic edges of the cornea. This vascularization is often so intense, as well as so delicate, in the character of the neoplastic vessels, as to give to the tissues a diffuse, dark red color, even to the degree of hiding the larger vessels from sight. In fact, so extreme may this become, that it will take careful observation not to be misled in assuming the existence of an excessive intra-corneal hemorrhage, completely filling the anterior chamber of the eye. *The latter never occurs, however.* As the processes increase in intensity, the yellowish center increases in extent, and always in a direction across the eye, from side to side, and then below the same will be seen a mass of intensely vascularized tissue, much swollen; but still the overlying tissues will retain their normal luster, or nearly so, while those over the yellowish center have become very thin and lusterless. (Fig. 2.) In many cases these abscesses rupture, and the contents at once escape. The rupture is invariably of the external tissues at first; the augmented amount of fluid in the anterior chamber exercising a perfectly equal pressure against Decemet's membrane, preventing a rupture in that direction. But when the abscess has been unusually extensive, the tissues forming its inner wall are too thin to resist this pressure, and a rupture soon follows, with escape of the aqueous humor, and prolapse of the lense, followed by the utter destruction of the complicated organ. In the majority of cases, this fatal termination does not occur, but from the ruptured edges of the external walls and base of such an abscess the development of granulation tissue begins, and extends across the cavity, completely filling it up, and projecting to a considerable degree beyond the level of the surrounding tissues; naturally, the size and shape of this mass will vary with the size and shape of the original abscess, and extent of the rupture. Such conditions have been illustrated in Figs. 3 and 4. From these granulations the previously mentioned vascularization may be seen extending in all directions towards the peripheries of the cornea. To one observing this disease for the first time, and before a complete examination of a severely complicated eye has been made, the most natural hypothesis would be, that there must also be very extreme complications of the internal portions of the organ. He would find himself severely mistaken, however. *Aside from an edematous condition of the iris, the internal portions of the eye remain absolutely normal.* Even the aqueous humor, while increased in quantity, remains as clear and pellucid as the clearest of distilled water, as I have tested in every case, by the careful withdrawing of the same, with a sterilized glass-barreled syringe. The most exact microscopical examination has failed to reveal the presence of a single leucocyte.

*Termination.*—Singularly to say, notwithstanding the apparent severity of the external lesions, with the exception of the rare cases in which complete rupture of the cornea with prolapsus of the lense occurs, there is, or, better perhaps, has been, an absolute return to normality, and complete re-acquisition of sight. Where no rupture has occurred, or where there has been no accumulation of pus, the first step towards restoration is a decrease in the caliber and number of the neoplastic vessels, with a clearance of the cornea at its peripheries, which slowly but surely extends towards the centers until the entire organ is again as transparent as could be desired. Where there has been a rupture, and the cavity caused thereby filled by granulation tissue, the same processes occur, and the same phenomena are seen, the vascularization decreasing and the granulation tissue becoming more and more anemic, and less and less prominent, until it finally entirely disappears, its place being at first represented by a yellowish-white, then white, and finally opaque, pearly spot, which eventually disappears entirely, the external epithelium having again completely covered the spot, and restitution is perfected in a manner seldom, if ever, seen in any process of wound-healing where the previous lesions have been of such apparent severity.

I will here remark that all these conditions have been followed, step by step, by microscopical studies made upon eyes removed at every distinct phase of the disease, but that it is my purpose to reserve their description for another paper, as well as some remarks as to a micro-organism present in the tissues. Cultivation attempts have thus far been futile, however.

*Nature.*—The extremely slow manner in which this disease extends over a herd of cattle, has been previously mentioned. No less remarkable has been the (in my experiments) absolute impossibility of intentional transmission of the disease from afflicted to healthy animals. For example, completely sterilized plugs of absorbent cotton were placed in the conjunctival sac (of a bull recently affected, but with a very profuse discharge,) until completely saturated. In one case, such plugs were placed within the same sac of the eyes of a healthy calf, while in another the cornea had been previously scarified with a sterilized lancet; notwithstanding the lids were held closed for five minutes in each case by attendants, *the results were absolutely negative.* The aqueous humor of the eye of a diseased calf having been withdrawn with a sterilized syringe, was injected into the anterior chamber of the eyes of a rabbit, and, as well as possible, into the tissues of the cornea of another, with the same unsatisfactory results. They do not, however, have any value as evidence against the conclusion that the disease is of a contagious character. For this speaks its appearance in a single individual at first, and its very gradual extension, while were it due to any specific, though external, cause, where all the animals are banded together, and hence the same treatment, a more general eruption would certainly occur.

*Prevention and Treatment.*—Complete isolation of the diseased from the healthy animals will cut off the extension of an outbreak with the most happy results.

All the treatment necessary is a dark place, and cloths constantly hanging over the eyes, kept wet with cold water all the time. This treatment lessens the severity of the disturbances, and hence tends to shorten the period of restitution.

The application of any washes or remedies inside the conjunctival sac is not only useless/but harmful, as the animals resist it all they can, and hence the danger of the introduction of irritating foreign material is increased, and even the endeavor to lift the lids, or handle the eye, must be looked upon as having an injurious tendency.

PATHO-BIOLOGICAL LABORATORY, LINCOLN, NEBRASKA.

## Figures and Tables

**Fig. I. Fig. II. Fig. III. Fig. IV. f1:**